# Supplementary material for: Decompensated MASH-Cirrhosis Model by Acute and Toxic Effects of Phenobarbital
Source: Cells. 2024 Oct 16;13(20):1707. doi: 10.3390/cells13201707 (PMC11505720; doi:10.3390/cells13201707)
Supplement: Supplementary file 1 [file cells-13-01707-s001.zip › cells-3145917-supplementary.pdf]

A) Hepatic hematoxylin and eosin staining of rats with ST Phenobarbital treatment at different timepoints

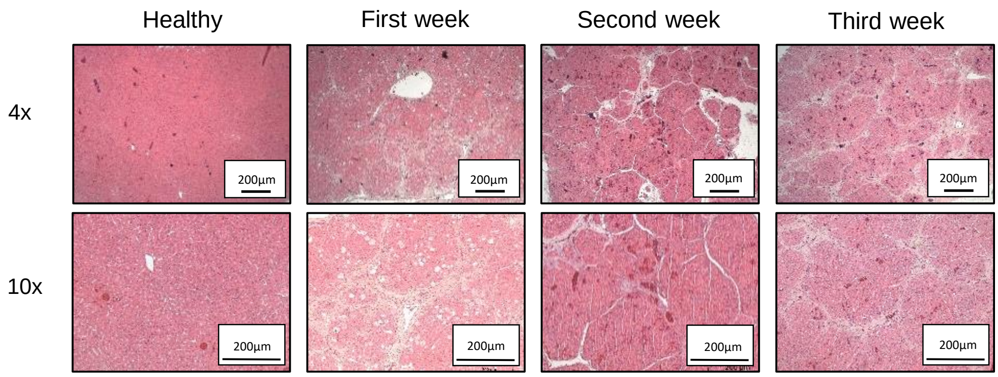

B) Hepatic αSMA staining of rats with ST Phenobarbital treatment at different timepoints

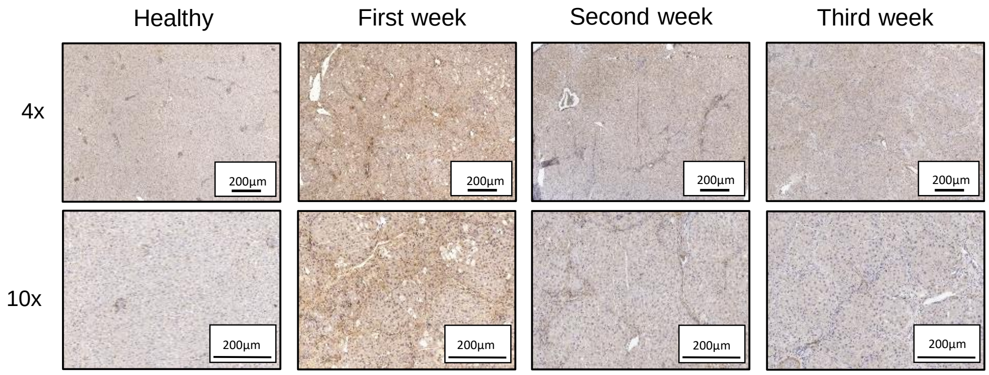

C) Hepatic pico sirius red staining of rats with ST Phenobarbital treatment at different timepoints

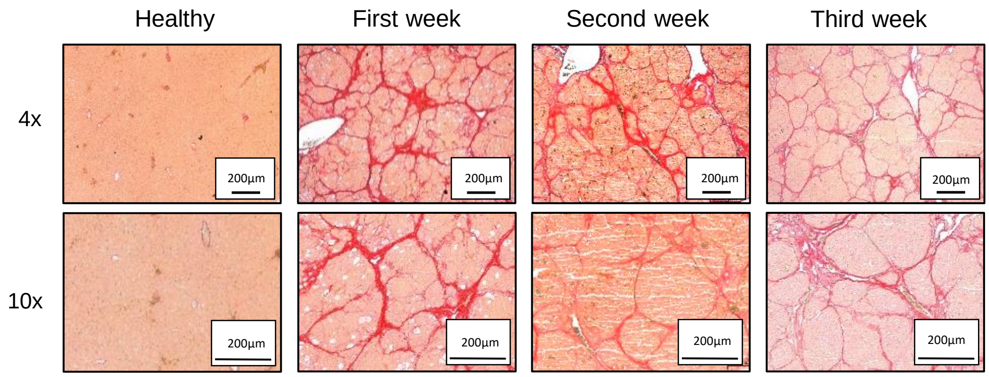

Figure S1: Characterization of Liver Fibrosis after ST treatment. Effect of carbon tetrachloride (CCl_4), CCl_4 + WD and Phenobarbital at different timepoints. Animals were treated with Phenobarbital (0.3 g/L) for 3 consecutive days in week 1, week 2 or week 3. Histological stainings of liver tissue of healthy, First week, Second week and third week ST treatment of Phenobarbital. Hematoxylin and esosin (H&E) in the livers of healthy, first week, second week and third week ST treated rats (A), α-smooth muscle actin (α-SMA) (B) and Pico Sirius red (C) stainings of the above mentioned rats.

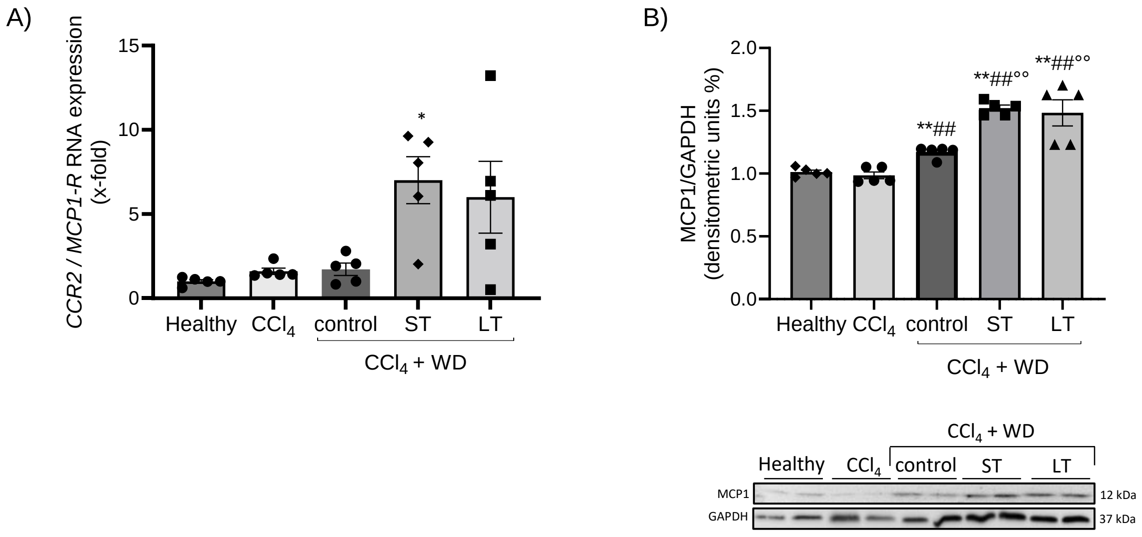

Figure S2: Hepatic inflammation. Effect of carbon tetrachloride (CCl_4), CCl_4 + WD and Phenobarbital in liver inflammation in SD rats. mRNA expression of C-C chemokine receptor type 2 (CCR2) (A) and protein levels of corresponding MCP1 (B) in liver tissues of healthy, CCl_4, control (CCl_4 + WD), ST and LT treated rats. (*/**p<0.05/p<0.005 vs. Healthy; ##p<0.005 vs. CCl_4; °°p<0.005 vs. Control).

Table S1: Antibodies

Name	Order Number	Company
α-SMA	ab5694-100	Abcam plc, Cambridge, UK
Col1a1	91144S	Cell Signaling Technology, Inc., MA, USA
GAPDH	sc-47724	Santa Cruz Biotechnology, Santa Cruz, CA, USA
SREBP1	ab28481	Abcam plc, Cambridge, UK
MCP1	Sc-28879	Santa Cruz Biotechnology, Santa Cruz, CA, USA

Table S2: TaqMan qPCR Assays

Name	Order Number	Company
α-SMA	Rn01759928	ThermoFisher Scientific, Darmstadt, GER
Col1a1	Rn00801649	ThermoFisher Scientific, Darmstadt, GER
GFAP	Rn01253033	ThermoFisher Scientific, Darmstadt, GER
EMR1	Rn01527631	ThermoFisher Scientific, Darmstadt, GER
IL6	Rn01410330	ThermoFisher Scientific, Darmstadt, GER
MCP1/CCL2	Rn00580555	ThermoFisher Scientific, Darmstadt, GER
MCP1-R/CCR2	Rn01637698	ThermoFisher Scientific, Darmstadt, GER
